# Nanogels of dual inhibitor-modified hyaluronic acid function as a potent inhibitor of amyloid β-protein aggregation and cytotoxicity

**DOI:** 10.1038/s41598-018-21933-6

**Published:** 2018-02-22

**Authors:** Zhiqiang Jiang, Xiaoyan Dong, Xin Yan, Yang Liu, Lin Zhang, Yan Sun

**Affiliations:** 1 Department of Biochemical Engineering and Key Laboratory of Systems Bioengineering of the Ministry of Education, School of Chemical Engineering and Technology, Tianjin University, Tianjin, 300072 China; 2 Department of Biology & Guangdong Provincial Key Laboratory of Marine Biotechnology, College of Science, Shantou University, Shantou, Guangdong 515063 China

## Abstract

Inhibition of amyloid β-protein (Aβ) aggregation is considered as a promising strategy for the prevention and treatment of Alzheimer’s disease. Epigallocatechin-3-gallate (EGCG) and curcumin have been recognized as effective inhibitors of Aβ aggregation. Herein, we proposed dual-inhibitor modification of hyaluronic acid (HA) to explore the synergistic effect of the two inhibitors. EGCG-modified HA (EHA) formed dispersed hydrogel structures, while EGCG-curcumin bi-modified HA (CEHA) self-assembled into nanogels like curcumin-modified HA (CHA). Thioflavin T fluorescent assays revealed that the inhibitory effect of CEHA was 69% and 55% higher than EHA and CHA, respectively, and cytotoxicity assays showed that the viability of SH-SY5Y cells incubated with Aβ and CEHA was 28% higher than that with Aβ and the mixture of EHA and CHA. These results clearly indicate the synergism of the two inhibitors. It is considered that the difference in the hydrophobicities of the two inhibitors made the bi-modification of HA provide a favorable CEHA nanostructure that coordinated different inhibition effects of the two inhibitors. This research indicates that fabrication of dual-inhibitor nanosystem is promising for the development of potent agents against Aβ aggregation and cytotoxicity.

## Introduction

Alzheimer’s disease (AD), the main kind of dementia, is an irreversible devastating neurodegenerative disorder characterized by a progressive loss of memory and other cognitive abilities^1,2^. Although the precise pathogenesis of AD is still uncertain, investigations in the past decades have put out several pathological hypotheses^3–5^. Among them, amyloid hypothesis is widely accepted, and it is believed that the abnormal accumulation, aggregation and deposition of amyloid β-protein (Aβ) contribute to the cerebral extracellular amyloid plaques that cause neurotoxicity^6–8^. Thus, inhibition of Aβ aggregation is considered as a promising strategy to fight against AD.

Till now, many different kinds of Aβ aggregation inhibitors have been reported, including small molecules^9–11^, peptides^12,13^, antibodies^14^ and nanoparticles (NPs)^15–17^. Most of the inhibitors work by binding or adsorbing Aβ, which affects the conformational changes of Aβ and/or blocking the protein interactions. Of the inhibitors, natural polyphenols such as epigallocatechin-3-gallate (EGCG) and curcumin have received extensive studies because they show safety and high efficiency in the inhibition of Aβ aggregation^18–20^. EGCG and curcumin have similar structures (Fig. S1 in Supplementary Materials) and their inhibitory mechanisms are similar to some degree. Their aromatic rings can interact with the aromatic residues in amyloidogenic proteins, preventing the π–π interaction and blocking the aggregation process^21,22^. Several literatures reported that both of them could change the pathway of Aβ aggregation and remodel Aβ fibrils^23,24^. However, there exist some differences between the effects of EGCG and curcumin on Aβ aggregation. It was reported that EGCG could directly bind to unfolded Aβ and promote the formation of unstructured non-toxic oligomers^25^, while curcumin could bind to Aβ due to its unique structure with two terminal phenyl groups and a rigid linker region between 8 and 16 Å in length^26^. EGCG could bind to the 12 important residues of Aβ_42_ (Phe4, Arg5, Phe19, Phe20, Glu22, Lys28, Gly29, Leu34-Gly37, and Ile41) and also form hydrogen bond with Aβ^27^, while curcumin was reported to bind to 12th and 17th to 21st residues of Aβ_42_^28^. Some researchers believed that curcumin could only bind to the fibrillar conformation, but not to shorter, aggregation-incompetent Aβ fragments^29^. These literature results suggest that EGCG and curcumin are both effective inhibitors of Aβ aggregation, but they work differently. Thus, it is speculated that there may be synergistic effects of these two inhibitors when they are put together in a nanosystem. Thus, this work was designed to fabricate a dual-inhibitor nanosystem with EGCG and curcumin to utilize their different effects and explore their potential synergistic effects.

It has been known that conjugating curcumin to the highly hydrophilic hyaluronic acid (HA) can increase curcumin solubility, and the curcumin-HA (CHA) conjugates can self-assemble into NPs via hydrophobic interactions mediated by the conjugated curcumin molecules^30,31^. In our previous work^32^, we have shown that CHA nanogels showed a size-tunable property depending on the substitution degree (SD) of curcumin (SD_C_). More importantly, it turned out that CHA inhibited Aβ fibrillogenesis and mitigated the amyloid cytotoxicity more efficiently than free curcumin, and that there existed an optimal SD_C_, at which the nanogels exhibited the best inhibitory effect. Based on the findings, a mechanistic CHA model was proposed. It suggested that besides the inhibitory effect of the conjugated curcumin, CHA nanogels provided additional functions: (1) the hydrogel network provided an isolation effect that could hinder the interactions between Aβ molecules; (2) hydrophobic binding of Aβ to curcumin and electrostatic repulsion between the bound Aβ and like-charged HA could stretch the conformation of Aβ monomers, thus leading to off-pathway aggregations. The latter was referred to as hydrophobic binding and electrostatic repulsion (HyBER) effect.

However, the research has revealed that CHA showed moderate increase in the inhibition effect as compared to free curcumin, and the inhibitory potency relied on whether the nanostructure of CHA was suitable for both isolation and HyBER effects. So, CHA of high SD showed lower inhibitory effect because its nanostructure was too compact for Aβ molecules to enter^32^. In order to address this issue, we have herein proposed to introduce a hydrophilic polyphenol inhibitor, EGCG, into the system to decrease the total hydrophobicity of the conjugate. It is expected that the different hydrophobic properties of EGCG and curcumin^33,34^ could help tune the nanostructures of the conjugates to reach more favorable isolation and HyBER effects. In addition to the improvement in nanostructure, it is also expected that the dual-inhibitor system could enhance the inhibitory effect on Aβ aggregation by incorporating the potential synergistic effects of EGCG and curcumin described above. In this article, we first presented the effect of EGCG-HA (EHA) as a new example of the inhibitor-HA conjugate system. Then, the bi-modified HA with EGCG and curcumin (CEHA) with a series of SD values of EGCG and curcumin was studied to explore the best combination of the two inhibitors. The nanostructure effects on Aβ aggregation were then studied. The bi-modified and mono-modified conjugates were compared and the synergistic effects of EGCG and curcumin on CEHA were discussed to provide insight into its working mechanism.

## Results

### Characterization of EHA and CEHA conjugates

The reactions for the synthesis of EHA and CEHA conjugates are illustrated in Fig. S1 in the Supplementary Materials. The purity of CEHA conjugate was ≥96%, as determined by reversed-phase high-performance liquid chromatography (RP-HPLC) (Fig. S2). The chemical structures of CEHA conjugates were analyzed using ^1^H-NMR (Fig. S3) and Fourier transform infrared spectroscopy (FTIR) (Fig. S4). The characteristic peaks of HA appeared at the range of methyl adjacent to carbonyl (δ = 1.85 ppm, [-COCH_3_]) and methylene/methine (δ = 3.2–3.8 ppm) (Fig. S3a). Successful synthesis of CEHA was confirmed by the peaks appearing from methylene and methine adjacent to benzene ring in EGCG (δ = 2.8–2.9 ppm, 2 H, PH-C*H*_2_-CO and δ = 5.45 ppm, 1 H, PH-C*H*-O), OCH_3_ groups in curcumin (δ = 3.82 ppm), the benzene ring in both EGCG and curcumin (δ = 6.5–7.1 ppm) and hydrogen atom adjacent to benzene ring and double bond in curcumin (δ = 7.60 ppm, 1 H, PH-C*H*=) (Fig. S3d). From the FTIR, some specific bonds and groups were identified at the wavenumber (cm^−1^) of 600–900 for benzene rings, 1300–1350 for C-O stretching frequency of ester linkage, 2100 broad band and 2800–3000 peaks for curcumin unsaturated hydrogen and the changes of band at 1100 and 3600 for the phenols (Fig. S4).

Substitution degrees of EGCG (SD_E_) were determined by measuring absorbance at 280 nm using a calibration curve (Fig. S5a,b). EHA has a similar spectrum as that of EGCG, so it was reasonable to assume that EGCG was conjugated onto the HA backbone considering HA has no absorbance at all in the wavelength range. The red shift of EHA was caused by the auxochromic effect of numerous hydroxyl groups and the ester linkage^30,31^. SD_E_ values of the three synthesized EHAs were 1.67, 4.63 and 12.70 (Table S1).

EGCG and curcumin are both polyphenol inhibitors of Aβ aggregation but have different hydrophobicities^33,34^. We have shown that CHA conjugates formed stable nanoparticles^32^. By contrast, dynamic light scattering analyses (Fig. S6) and transmission electron microscopy observations (Fig. S7) revealed that EHA formed less single NPs but more large dispersed hydrogels. This is considered due to the lower hydrophobicity of EGCG than curcumin. Therefore, introduction of curcumin to EHA led to the synthesis of CEHA that formed stable NPs (Fig. S8).

**Figure 1:**
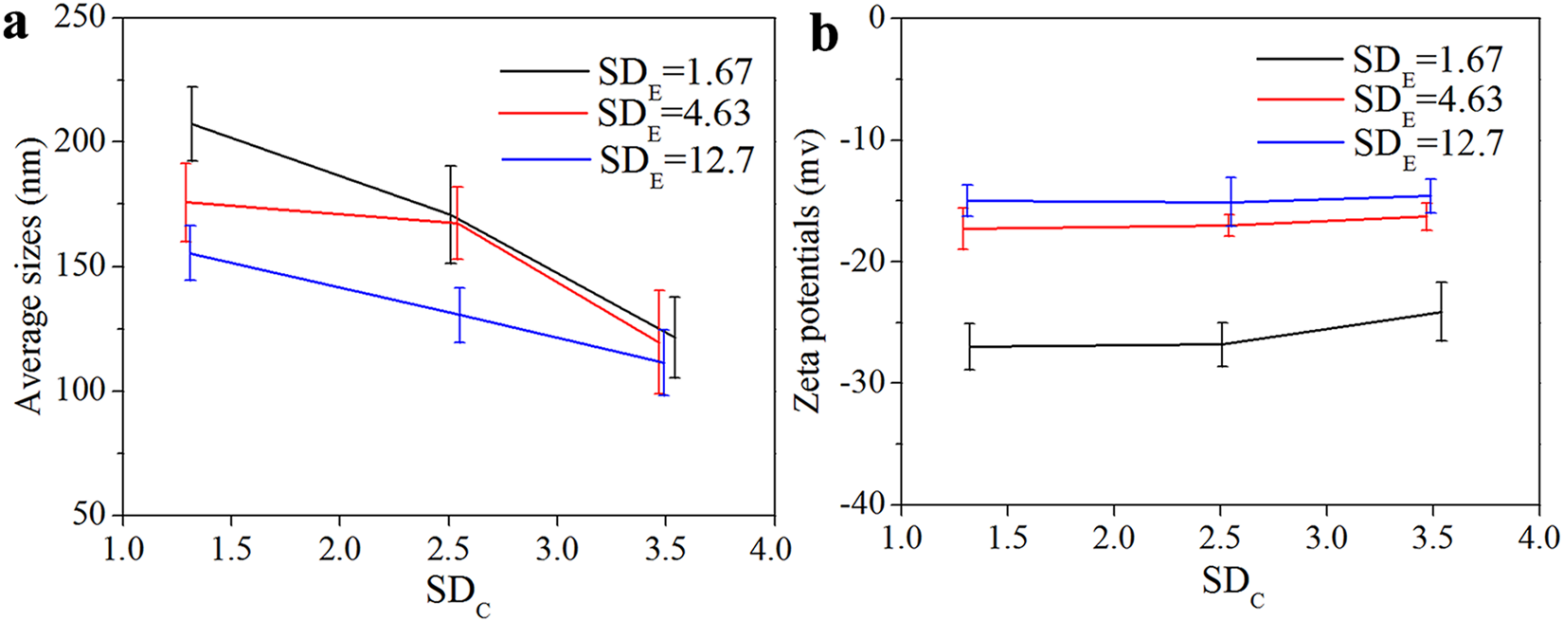
Average hydrodynamic sizes (**a**) and zeta potentials (**b**) of CEHA NPs at different SD_E_ as a function of SD_C_ in the PBS solution at 37 °C. The data were the average values of the data determined with three samples and the error bars represent the standard deviations.

Three CEHAs at each SD_E_ were then synthesized (Table S2). The SD_C_ values of the CEHAs were determined by measuring the absorbance at 440 nm using a calibration curve (Fig. S5c,d). The hydrodynamic size (diameters) of the CEHAs in the PBS solution decreased as SD_C_ or SD_E_ increased (Fig. 1a). The mean sizes of the CEHAs were more sensitive to SD_C_ than to SD_E_, due to the higher hydrophobicity of curcumin. There was no discernible difference in the zeta potentials of CEHAs as SD_C_ changed (Fig. 1b).

In the following discussion, the conjugate concentration used below for EHA, CHA or CEHA is described by the concentration of inhibitor, EGCG or curcumin, in the conjugate, to evaluate the conjugate inhibitory effect and compare with free inhibitors. Namely, the EGCG/curcumin concentration in the supplied CEHA was adjusted to match the concentration of the specific free molecule in solution.

### Elevated inhibitory effect of EHA on Aβ_42_ aggregation

**Figure 2:**
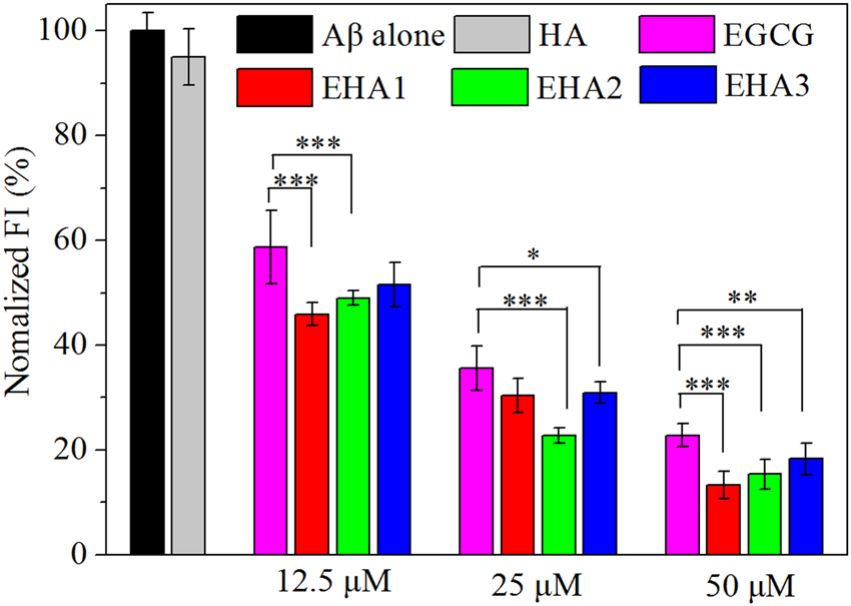
ThT fluorescence intensities of Aβ_42_ (25 μM) incubated for 48 h with HA, free EGCG and the three EHAs of different EGCG SD_E_ values. HA concentration was 2 mg/mL. EHA concentration is expressed as the equivalent concentration of EGCG carried in the conjugate. Each condition of incubation was conducted in triplicate in three Eppendorf tubes and the average value is reported with standard deviation. ***p < 0.001, **p < 0.01 and *p < 0.05 compared to free EGCG groups.

Figure 2 shows that HA had no remarkable influence on the thioflavin T fluorescent intensity (ThT FI) of Aβ_42_ at high concentrations. However Aβ_42_ incubated with EHA hydrogels had slightly lower ThT FI value than that incubated with free EGCG at the same EGCG concentration, demonstrating that the inhibitory effect of EGCG on Aβ_42_ aggregation was improved by conjugating onto HA. It also shows a dose-dependent effect of EHAs on Aβ_42_ aggregation. The results demonstrate that the HA modification improved the inhibitory effect of the polyphenol on Aβ_42_ aggregation, and it is considered that the CHA model proposed previously also works to some extent for the dispersed EHA hydrogel structures^32^.

### Elevated inhibitory effect of CEHA on Aβ_42_ aggregation

**Figure 3:**
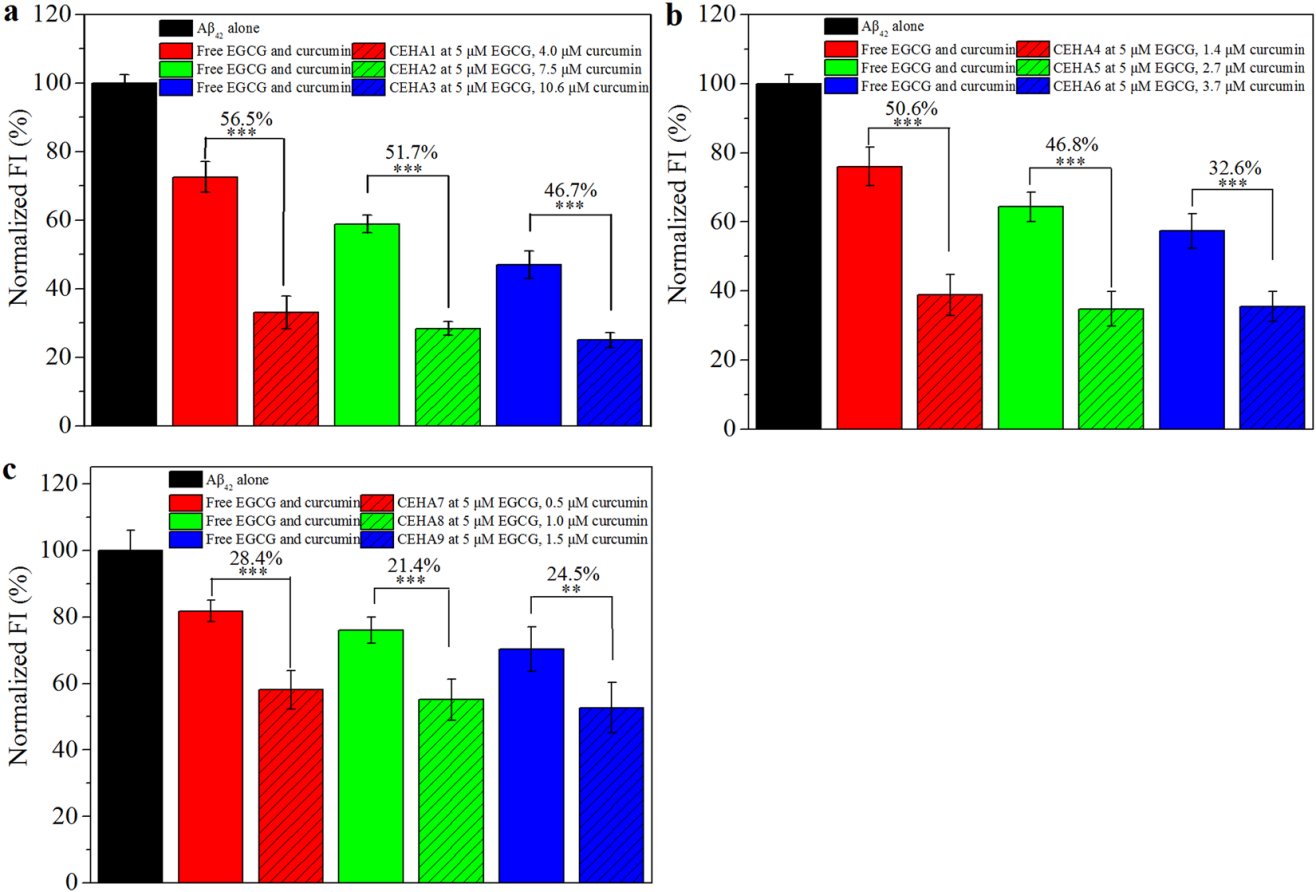
ThT FI of Aβ_42_ incubated for 48 h with different CEHAs by comparison with the mixture of free EGCG-curcumin at the same inhibitor concentrations at each group. CEHA concentration is expressed as the equivalent concentration of free EGCG and curcumin carried in the conjugates (CEHA mass concentrations were 0.016–0.12 mg/mL). EGCG concentration was fixed at 5 μM with varying curcumin concentrations. (**a**) CEHA1-3 (SD_E_ = 1.67); (**b**) CEHA4-6 (SD_E_ = 4.63); (**c**) CEHA7-9 (SD_E_ = 12.7). Each condition of incubation was conducted in triplicate in three Eppendorf tubes and the average value is reported with standard deviation. The figures in percentage above the columns stand for the RPs of the ThT FI for the CEHA groups (slash bars) as compared to those for the corresponding free EGCG-curcumin groups (solid bars). ***p < 0.001 and **p < 0.01 compared to the free inhibitor groups.

ThT FI of Aβ_42_ aggregation was investigated to examine the inhibitory effect of CEHA. At first, EGCG concentration was fixed at 5 μM and curcumin concentration varied from 0.5 to 10.6 μM for the first nine CEHA nanogels listed in Table S2 (CEHA mass concentration varied from 0.016 to 0.12 mg/mL for the different CEHAs to keep EGCG concentration unchanged). The ThT FI values of Aβ_42_ incubated with the nine CEHAs for 48 h are shown in Fig. 3, and the control groups were set as the free EGCG-curcumin mixture with the same EGCG/curcumin concentrations as the corresponding CEHA group. The figure over a column represents the reducing percentage (RP) of ThT FI for the CEHA as compared to that for the free EGCG-curcumin mixture. In each subfigure in Fig. 3, the CEHAs had the same SD_E_ but different SD_C_ values. Clearly, CEHAs showed higher inhibitory effect than the free inhibitor mixtures, because they decreased the ThT FI of Aβ_42_ aggregation by over 20% to 50% as compared to the free EGCG-curcumin groups. In general, the RP decreased with increasing SD_E_ as well as SD_C_. For example, the RP for CEHA1-3 decreased from 56.5% to 46.7% with the increase in SD_C_. It is considered that introducing more curcumin into the EHA backbone resulted in more compact nanostructure of CEHA, similar to the case of CHAs^32^, which compromised the inhibitory effect of the nanogels. By judging from the RP values, it is obvious that CEHA1-3 with the lowest SD_E_ (Table S2) were more efficient than the other six, indicating that SD_E_ was also important in influencing the CEHA nanostructure that contributed to the inhibitory effect of the CEHA conjugates.

When fixing curcumin concentration at 5 μM with varying EGCG concentrations (2.4–48 μM) for CEHA (CEHA mass concentration varied from 0.06 to 0.15 mg/mL for the different CEHAs to keep curcumin concentration unchanged), similar results were obtained (Fig. S9). However, in comparison of Fig. S9 with Fig. 3, it is clear that the RPs at lower curcumin and higher EGCG concentrations (Fig. S9) were much lower than those at lower EGCG and higher curcumin concentrations (Fig. 3). This indicates that the enhancing effect of the bi-conjugation of HA become less significant for the combination with higher EGCG concentrations.

**Figure 4:**
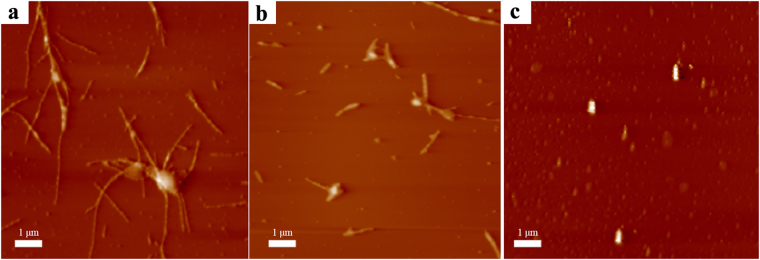
The AFM images of Aβ_42_ incubated for 48 h. (**a**) 25 μM Aβ_42_ alone; (**b**) 25 μM Aβ_42_ with a mixture of 5 μM EGCG and 4 μM curcumin; (**c**) 25 μM Aβ_42_ with 0.12 mg/mL CEHA1 nanogel (containing 5 μM EGCG and 4 μM curcumin).

The aggregation morphologies of Aβ_42_ incubated with different inhibitor systems were observed with atomic force microscopy (AFM). Figure 4a shows that Aβ_42_ alone formed mature fibrils of several micrometers in length. Incubation with free EGCG and curcumin mixture resulted in fewer fibrous aggregates (Fig. 4b). However, it is seen that few fibrils but spherical aggregates were observed in the presence of CEHA1 (Fig. 4c), indicating that the CEHA nanogels altered the structure and morphologies of Aβ_42_ aggregates.

Finally, cell viability assays were performed to examine the detoxification effect of CEHA nanogels. EGCG was little cytotoxic but curcumin was slightly toxic at 25 μM (Fig. S10a). In comparison, little cytotoxicity of the nine CEHA nanogels at 25 μM curcumin was observed (Fig. S10b), suggesting the biocompatibility of the CEHA nanogels.

**Figure 5:**
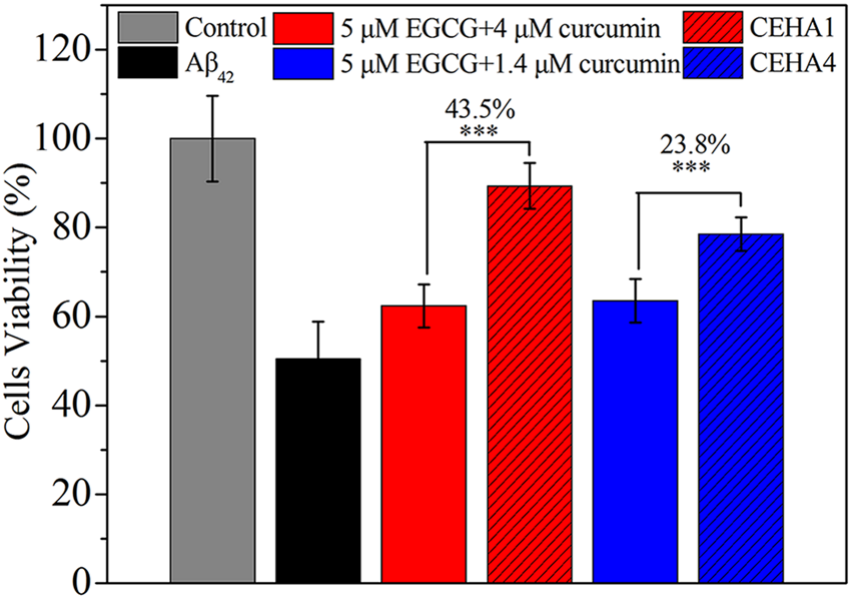
Cell viability of SH-SY5Y cells incubated with Aβ_42_ aggregates obtained by pre-incubation of Aβ_42_ with free EGCG-curcumin groups (solid bars) or CEHA (slash bars). All the samples had 5 μM EGCG. Each CEHA group contained the same EGCG and curcumin concentrations as the corresponding free EGCG-curcumin group. Each condition was conducted in sextuplicate in six wells of 96-well plate and the average value is reported with standard deviation. ***p < 0.001 compared to the free inhibitor groups.

Two CEHA conjugates, CEHA1 and CEHA4, which showed better inhibition performance (lower FIs and higher RPs) as evidenced in Fig. 3, were chosen for cell viability assays. As shown in Fig. 5, the cell viability in the group treated with PBS was set as 100%. CEHA1 (containing 5 μM EGCG and 4 μM curcumin) improved cell viability by 43.5% and CEHA4 (containing 5 μM EGCG and 1.4 μM curcumin) improved cell viability by 23.8%, as compared to the corresponding free inhibitor systems. With the CEHA1 containing only 5 μM EGCG and 4 μM curcumin, the cell viability increased to 89%, about 77% higher than the Aβ_42_ alone group (cell viability, 50.5%). CEHA1 presented higher performance than CEHA4. Higher curcumin concentration in CEHA1 might be part of the cause, but considering that such a low curcumin concentration does not provide significant inhibition effect^35,36^, difference in the nanostructures of the two nanogels was most likely to be responsible for the significant effect on the cell viability results.

### Nanostructure effect

**Figure 6:**
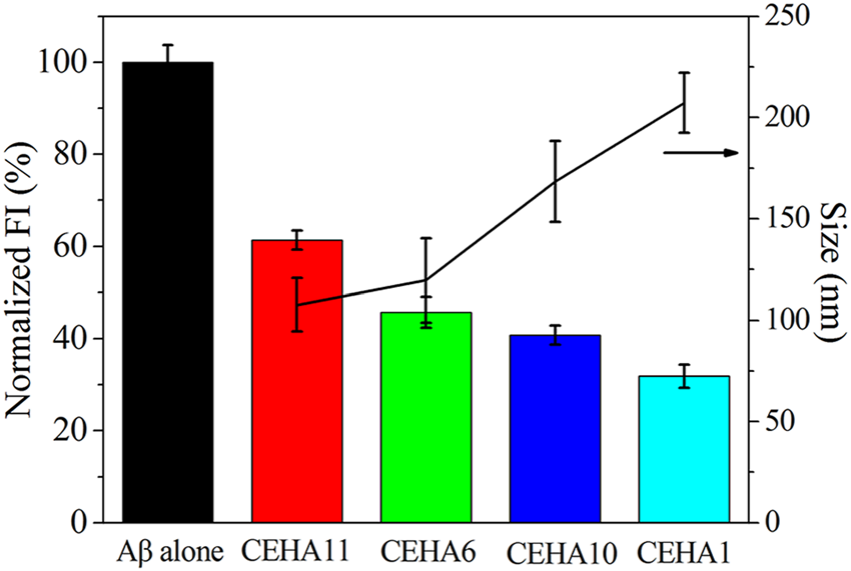
ThT FI of Aβ_42_ incubated for 48 h with the four CEHAs with almost the same SD_E_/SD_C_ ratio (1.3 ± 0.04). All CEHAs contains 5 μM EGCG and 3.8 ± 0.2 μM curcumin. Each condition of incubation was conducted in triplicate in three Eppendorf tubes and the average value is reported with standard deviation.

Thus, to better understand the relationship between the nanostructure and the inhibitory effect, we synthesized two more CEHA conjugates, CEHA10 and CEHA11 (Table S2). CEHA1, CEHA6, CEHA10 and CEHA11 have almost the same SD_E_/SD_C_ ratio (1.30 ± 0.04). Thus, we can design an inhibition experiment with these four CEHAs containing both EGCG and curcumin at the same concentrations (the mass concentration of CEHAs varied from 0.04 to 0.12 mg/mL to keep EGCG and curcumin concentrations unchanged) (Fig. 6). The average hydrodynamic size (diameter) of the CEHAs increased as total SD decreased (CEHA1 < CEHA10 < CEHA6 < CEHA11 in total SD), because the nanostructures became less compact at lower total SD values as discussed above. Interestingly, the ThT FI of Aβ_42_ incubated with the CEHAs also decreased as the average CEHA hydrodynamic size increased (Fig. 6). For example, CEHA1 reduced the ThT FI about 48.6% more than CEHA11 did. Since EGCG and curcumin concentrations (5 μM EGCG and 3.8 ± 0.2 μM curcumin) were almost the same for the four conjugates, this clearly indicates that the CEHA nanostructure significantly influenced the inhibitory effect of the conjugates. It is considered that in the four conjugates, CEHA1 had the most suitable SD that led to the formation of the most favorable nanostructure for Aβ_42_ to enter into and to interact with the inhibitors. This allowed CEHA1 to make full use of the conjugated inhibitors and coordinated the isolation and HyBER effects^32^ to achieve the highest inhibitory effect among the four conjugates.

### Synergistic effect

The mono-modified EHAs and CHAs both improved the effect of the polyphenol inhibitors based on the self-assembled structures of the conjugates. Considering that EGCG and curcumin have different hydrophobicities^33,34^ and different inhibition mechanisms in molecular level^25–29^, it is expected that a bi-modified CEHA with a suitable EGCG/curcumin ratio could form a favorable nanostructure for inhibiting Aβ_42_ aggregation and display a synergistic effect of EGCG and curcumin.

**Figure 7:**
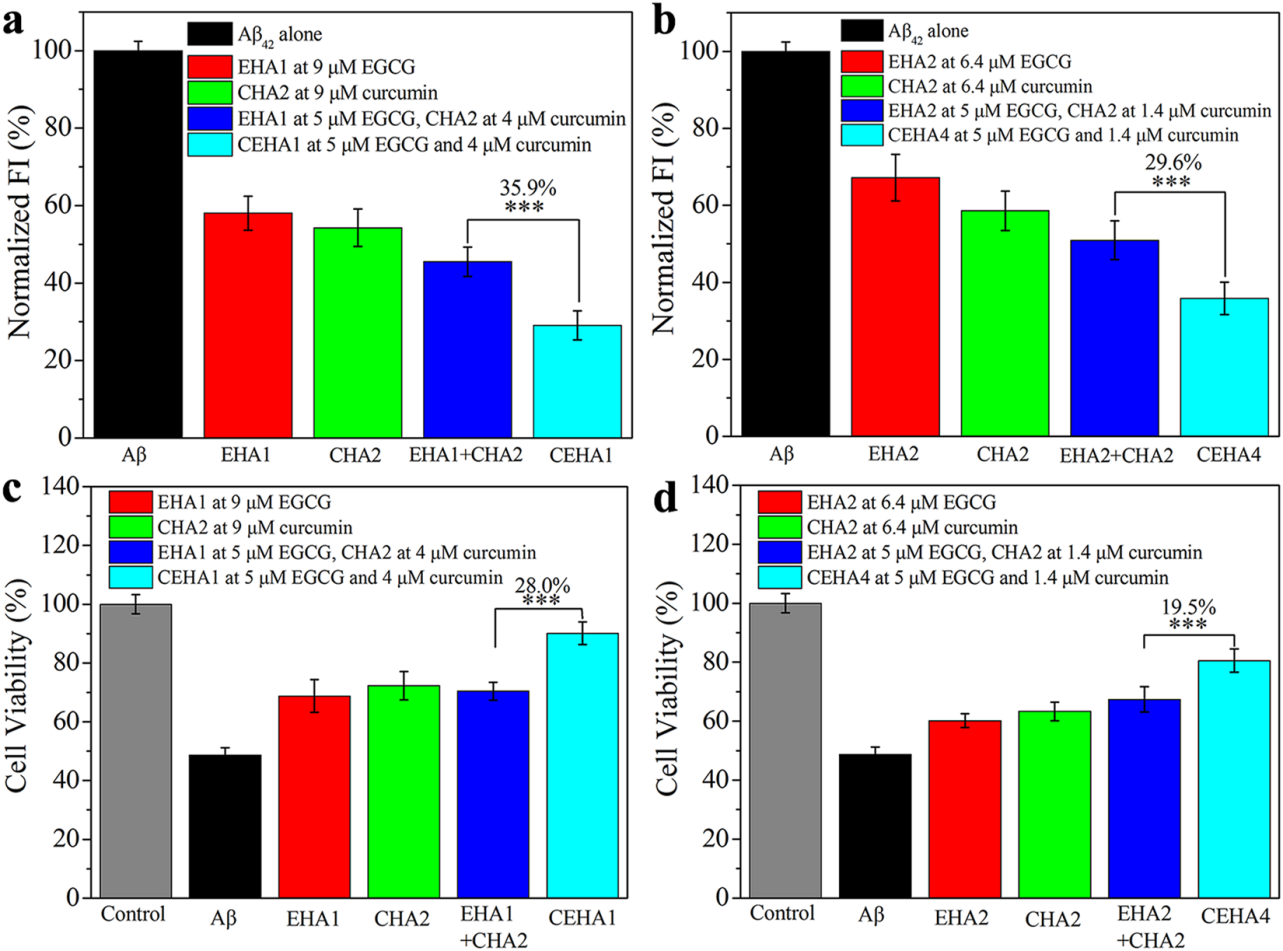
(**a**,**b**) Comparison of the ThT FI values of Aβ_42_ (25 μM) incubated for 48 h with EHA, CHA, mixture of EHA and CHA, and CEHA at the same total conjugated polyphenol concentration 9 μM in (**a**) and 6.4 μM in (**b**). Each condition of incubation was conducted in triplicate in three Eppendorf tubes and the average value is reported with standard deviation. (**c**,**d**) Cell viability of SH-SY5Y cells incubated with Aβ_42_ aggregates obtained by pre-incubation of Aβ_42_ with EHA, CHA, mixture of EHA and CHA and CEHA at the same total conjugated polyphenol concentration 9 μM in (**c**) and 6.4 μM in (**d**). Each condition was conducted in sextuplicate in six wells of 96-well plate and the average value is reported with standard deviation. (**a**) and (**c**) Comparison of EHA1, CHA2, mixture of EHA1 (SD_E_ = 1.67) and CHA2 (SD_C_ = 1.32), and CEHA1 with the same SD_E_ as EHA1 and SD_C_ as CHA2; (**b**) and (**d**) Comparison of EHA2, CHA2, mixture of EHA2 (SD_E_ = 4.63) and CHA2 (SD_C_ = 1.32), and CEHA4 with the same SD_E_ as EHA2 and SD_C_ as CHA2. ***p < 0.001 compared to the mono-modified EHA and CHA mixture.

To verify the synergistic effect, we compared the difference between a bi-modified CEHA and a mono-modified EHA or CHA at the same (total) inhibitor concentrations. Figure 7a shows the ThT FI data of Aβ_42_ incubated with EHA, CHA, the mixture of EHA and CHA, and CEHA at a total inhibitor concentration of 9 μM. It is clear that Aβ_42_ incubated with CEHA1 (SD_E_ = 1.67, SD_C_ = 1.32) displayed the lowest FI, and its FI was 35.9% lower than that with the mixture of EHA1 and CHA2. CEHA4 showed the same tendency (Fig. 7b). Cell viability assays at the same (total) inhibitor concentrations exhibited similar results: the cell viability with CEHA1 was 28.0% higher than that with the mixture of EHA1 and CHA2, and the cell viability with CEHA4 was 19.5% higher than that with the mixture of EHA2 and CHA2 (Fig. 7c,d). CEHA1 and CEHA4 increased the cell viability to 90.1% and 80.5%, respectively, which were respectively 85.1% and 65.4% higher than the Aβ_42_ alone group.

The synergistic effect of conjugated EGCG and curcumin in CEHAs on inhibiting Aβ_42_ aggregation was further studied by ThT fluorescent assays. Here, we introduced a synergy factor, *SF*, defined as^37^:

1 $$SF=\frac{100 \% -{R}_{AB}}{100 \% -{R}_{A}\times {R}_{B}}\,$$

where *R*_A_ and *R*_B_ are the remaining ThT FI for the mono-inhibitor system and *R*_AB_ is the remaining ThT FI for the dual-inhibitor system. *SF* > 1 means a synergism of the two inhibitors, whereas *SF* < 1 means antagonism of the two inhibitors.

**Figure 8:**
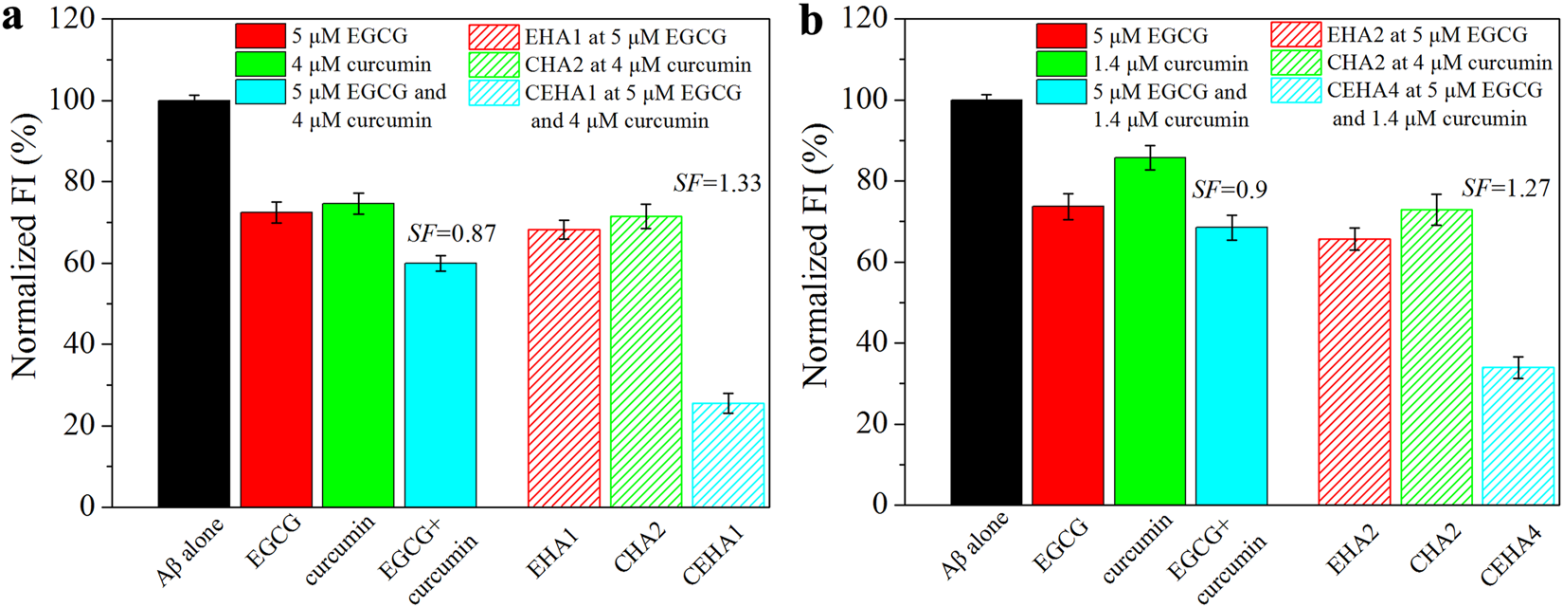
(**a**) Determination of the *SF* value for CEHA1: ThT FI of Aβ_42_ alone and incubated with EHA1 at 5 μM EGCG (red slash bars), CHA2 at 4 μM curcumin (green slash bars) and CEHA1 at 5 μM EGCG and 4 μM curcumin (cyan slash bars). (**b**) Determination of the *SF* value for CEHA4: ThT FI of Aβ_42_ alone and incubated with EHA2 at 5 μM EGCG (red slash bars), CHA2 at 1.4 μM curcumin (green slash bars) and CEHA4 at 5 μM EGCG and 1.4 μM curcumin (cyan slash bars). Aβ_42_ incubated with free EGCG and curcumin at the same concentrations are also reported as controls in the two figures. Each condition of incubation was conducted in triplicate in three Eppendorf tubes and the average value is reported with standard deviation.

The *SF* values for the two representative dual-inhibitor conjugates, CEHA1 and CEHA4, were determined by ThT fluorescent assays (Fig. 8) to be 1.33 and 1.27, respectively. As controls, free EGCG-curcumin showed an antagonistic effect with an *SF* value of about 0.9. The results indicate that only the conjugated EGCG and curcumin in the CEHA nanogels had significant synergistic functions. In other words, the dual-conjugation onto HA was the cause for the synergistic effect.

Effects of CEHA on the kinetics of Aβ_40_ aggregation was studied by measuring the aggregation dynamics. The growth curves were normalized using a sigmoidal fit equation^38^,

2 $$y={y}_{0}+\frac{{y}_{max}-{y}_{0}}{1+exp(-(t-{t}_{1/2})k)}$$

where *y* is the ThT FI at time *t*, *y*_0_ and *y*_max_ are the minimum and maximum ThT FI, respectively, *t*_1/2_ is the time when the ThT FI reaches half the maximum intensity, and *k* is the apparent first-order aggregation constant. From the fitting the lag phase time (*T*_lag_) could be calculated from^38^,

3 $${T}_{{\rm{lag}}}={t}_{1/2}-2/k$$

Fig. S11 shows the fitting of Eq. (2) to the normalized dynamic data. The apparent first-order aggregation constant *k* value and lag phase time *T*_lag_ determined from Eq. (3) are listed in the Table 1. EGCG did not affect the *k* value although it decreased the plateau ThT FI of 25 μM Aβ_40_ by 40% at 9 μM. Curcumin decreased the *k* value slightly and prolonged the *T*_lag_ of 25 μM Aβ_40_ by 40% at 9 μM. This further demonstrated the different working mechanisms of EGCG and curcumin. As for the nanogel (EHA, CHA or CEHA) containing the same concentration of EGCG or curcumin, it remarkably decreased the *k* value (e.g. CEHA1 decreased *k* from 0.68 to 0.16) and also prolonged the *T*_lag_. Considering that the primary nucleation rate can be reflected by the *T*_lag_ and the elongation rate and secondary nucleation rate by the *k* value^39^, it is clear that the nanogels enhanced the effects of EGCG and curcumin on the elongation and secondary nucleation processes.

**Table 1:** Kinetic parameters of Aβ_40_ aggregation^a^.

Inhibitors	*k* (h^−1^)	*t*_1/2_ (h)	*T*_lag_ (h)	Plateau ThT^b^ FI (%)
None	0.68 ± 0.03	21.0 ± 1.3	18.5 ± 1.2	100 ± 9.2
EGCG	0.64 ± 0.04	24.0 ± 0.8	20.9 ± 0.6	59.6 ± 4.4
Curcumin	0.55 ± 0.11	29.0 ± 1.6	25.3 ± 2.2	75.6 ± 4.6
EGCG + Curcumin	0.63 ± 0.09	28.3 ± 1.6	25.1 ± 1.9	80.1 ± 7.0
EHA	0.43 ± 0.05	28.7 ± 2.0	24.0 ± 2.5	62.1 ± 3.9
CHA	0.30 ± 0.06	37.7 ± 1.9	30.9 ± 3.0	44.0 ± 8.8
CEHA1	0.16 ± 0.02	40.8 ± 2.5	28.4 ± 3.2	33.8 ± 6.9

^a^Data represent average values of six replica of Aβ_40_ aggregation dynamics and the standard deviations.

^b^Steady-state ThT FI values. The value without any inhibitor was set as 100% (first row) and the others were normalized by comparison to this value.

## Discussion

It has been recognized that in on-pathway fibrillation, Aβ monomers aggregate to amyloid fibrils via several metastable oligomers after transition into β-rich conformations^40–43^. Previous work has revealed that CHA nanogels might provide an isolation effect to segregate the Aβ molecules and a HyBER effect that disturbed the conformation of the bound Aβ molecules^32^. These two effects were influenced by the conjugate nanostructure (Fig. 6). SD_C_ is a key factor influencing the conjugate nanostructure (Fig. 1a). When the SD_C_ was high, the nanostructure would become too compact, preventing Aβ from entering the nanogels. When the SD_C_ was very low, the nanostructure would become dispersed as in the case of EHA gel (Figs S6 and S7), which would weaken the isolation effect of the network. Because of the low hydrophobicity of EGCG, the EHA hydrogels should have loose structures and wide networks (Fig. S7). However, CEHA contained both EGCG and curcumin and the strong hydrophobicity of curcumin stabilized the nanostructures (Fig. S8). The presence of EGCG in CEHA would prevent the nanostructure from becoming too compact for Aβ to enter at higher SD values. In this way, conjugated EGCG could provide sufficient polyphenol inhibitor inside the nanostructure for binding Aβ. Thus, CEHA could not only exhibit favorable nanostructure (Fig. 6) that coordinated the isolation and HyBER effects, but also provided high conjugated inhibitor (including curcumin and EGCG) concentration that could function effectively inside the nano-conjugates. This is the synergistic effects of EGCG and curcumin in the bi-conjugated nanosystem as compared to EHA or CHA (Figs 7 and 8 and Table 1). The different hydrophobicities of EGCG and curcumin and suitable SD provided a favorable nanostructure for Aβ penetration and isolating them from each other. The HyBER effect of nanogels might influence the aggregation process of Aβ to decrease the *k* value and prolong *T*_lag_ (Table 1) and then enhance the effects of conjugated EGCG and curcumin on the elongation and secondary nucleation processes of Aβ_40_. Consequently, the favorable nanostructure of CEHA and the synergistic effect of the two inhibitors in the nanogels led to the potent inhibition on the cytotoxicity of Aβ aggregates at low total inhibitor concentrations (Figs 5, 7c,d).

In conclusion, this research has designed bi-modification of HA with EGCG and curcumin to develop a potent nano-inhibitor on Aβ aggregation and cytotoxicity. This design was to explore a potential synergistic effect of the two inhibitors in the conjugates because EGCG and curcumin are both effective inhibitors of Aβ aggregation, but they work differently. The synergistic effect has been proved by extensive inhibition experiments and a synergy factor over 1.3 was observed. It was found that the introduction of EGCG to the curcumin-modified HA could modulate the nanostructure of the self-assembled hydrogels. Therefore, there was a suitable substitution degrees of EGCG and curcumin, at which the CEHA could not only provide sufficient conjugated polyphenol inhibitors that functioned synergistically inside the nano-conjugates, but also provide a favorable nanostructure that effectively influenced the aggregation process of Aβ. The research indicates that fabrication of a dual-inhibitor nanosystem is promising for the development of a potent agent against Aβ aggregation and cytotoxicity.

## Methods

### Chemicals and reagents

Curcumin (98%), EGCG (95%), hexafluoroisopropanol (HFIP), 3-(4,5-dimethylthiazol-2-yl)-2,5-diphenyltetrazolium bromide (MTT), thioflavin T (ThT), 1, 3-dicyclohexylcarbodiimide (DCC) and 4-dimethylaminopyridine (DMAP) were obtained from Sigma-Aldrich (St. Louis, MO, USA). Hyaluronic acid sodium salt (HA) (MW >1 × 10^6^, 95%) was purchased from Aladdin (Shanghai, China). Dimethyl sulfoxide (DMSO) was purchased from Daji Huagong (Tianjin, China). Aβ_42_ and Aβ_40_ were received from GL Biochem (Shanghai, China). SH-SY5Y cells were from the Cell Bank of the Chinese Academy of Sciences (Shanghai, China). Dulbecco’s Modified Eagle Medium/Ham’s F-12 (DMEM/F12) and fetal bovine serum (FBS) were obtained from Invitrogen (Carlsbad, CA, USA). Other chemicals were all of the highest purity available from local sources. Deionized water was used for all solution preparations.

### Synthesis of EHA, CHA and CEHA conjugates

EHA and CEHA conjugates were synthesized according to the methods reported previously (Fig. S1c)^30–32^. Briefly, HA and EGCG/curcumin were dissolved in water/DMSO system (1:1 v/v) with DCC and DMAP as catalysts. The reaction was carried out at 65 °C for 6 h, and the resulting products were collected by dialysis with a membrane of molecular weight cut off of 10–14 kDa against DMSO for two days and then against deionized water for another three days to remove non-reacted substances and byproducts. The initial EGCG/HA in the reaction was tuned to prepare three EHA conjugates with different SD_E_.

CEHA conjugates were synthesized from EHA conjugates by further reaction with curcumin of different concentrations under the same reaction condition described above. The products were also recovered by the same procedure.

### Characterization of the conjugates

The purity of CEHA conjugates was analyzed by RP-HPLC on Agilent 1100series (Agilent Technologies, Santa Clara, CA) with a Waters C4 reversed-phase column (Waters, Milford, MA, USA) with UV-visible detections at 280 and 440 nm. The purified CEHA conjugates were then characterized by FTIR (Nicolet 6700, Nicolet, USA) and nuclear magnetic resonance spectroscopy (^1^H NMR) (Varian Inova, Varian Medical System, Inc., CA, USA).

The conjugates were then characterized by UV-visible spectroscopy. The SD_E_ and SD_C_ in the conjugates were calculated by the absorbance measurement at 280 nm and 440 nm, respectively, by using their calibration curves respectively prepared with free EGCG and curcumin^29,44^. The SD_E_ or SD_C_ was defined as the number of EGCG or curcumin molecules per hundred HA monomer units.

The morphologies and structures of EHA and CEHA self-assembled NPs were observed by transmission electron microscopy of model JEM-2100F (JEOL, Tokyo, Japan). The sample was prepared by dropping 10 μL solutions onto a carbon-coated copper grid and air-dried. The hydrodynamic size (diameter) of CEHA was determined by dynamic light scattering (DLS) by using Zetasizer Nano (Malvern Instruments, Worcestershire, UK) at 25 °C with the backscattered angle detection at 173°. The surface zeta potentials of the NPs were measured using the same equipment.

### Preparation of Aβ monomer solutions

Aβ_42_ and Aβ_40_ monomer solutions were prepared as described earlier^32,43^. The Aβ_42_ and Aβ_40_ protein sample was dissolved in HFIP and sonicated for 5 min in ice bath. It was then centrifuged at 16,000 g for 30 min at 4 °C. The upper 75% of the centrifugation supernatant was taken and frozen to −80 °C and HFIP was removed using a vacuum freeze drier (Labconco, MO, USA). The pre-treated Aβ_42_ and Aβ_40_ monomer sample was stored at −20 °C. Prior to use, the Aβ_42_ and Aβ_40_ monomer sample was dissolved in 20 mM NaOH and sonicated for 5 min in ice bath. It was then diluted to a PBS solution (100 mM phosphate buffer plus 10 mM NaCl, pH 7.4) with or without an inhibitor. This solution was used for the following inhibition studies of Aβ aggregation and cytotoxicity.

### ThT fluorescent assay

ThT is a widely used probe detecting amyloid fibrils or Aβ aggregates^45–47^. Aβ_42_ solutions (25 μM or 0.11 mg/mL) prepared as described above were incubated with or without an inhibitor by continuous shaking at 150 rpm in an air bath of 37 °C. Each condition of incubation was conducted in triplicate in three Eppendorf tubes and the average value is reported with standard deviation. The ThT FI was measured at different time intervals using fluorescence spectrometer of PE LS-55 (Perkin Elmer, MA, USA) at 25 °C with a slit width of 5 nm and excitation and emission at 440 and 480 nm, respectively. Before each measurement, 150 μL samples were withdrawn at different time intervals and mixed uniformly with 3 mL ThT solutions (25 μM ThT in 100 mM phosphate buffer, pH 7.4). The ThT FI of the samples without Aβ_42_ protein was subtracted as background from each read with Aβ_42_ protein. Student’s t-test (unpaired parametric test) was calculated for statistical comparisons to analyze the variance and p < 0.05 or less was considered to be statistically significant.

The kinetics of Aβ_40_ aggregation was monitored by *in situ* ThT fluorescent assays in a microplate reader (TECAN Infinite, Salzburg, Austria). Aβ_40_ was adopted in the kinetic assays because it has a distinct lag phase for investigating the nucleation process. By contrast, Aβ_42_ exhibits fast aggregation kinetics and in most cases no obvious lag phase could be observed^48,49^.

### Observation of Aβ_42_ aggregates

AFM is widely used to observe the morphologies of oligomeric structures, aggregates and mature amyloid fibrils^50,51^. The morphologies of Aβ_42_ aggregates were observed by atomic force microscopy of model CSPM 5500 (Benyuan, Guangzhou, China). An AFM sample was prepared by dropping 10 μL of 25 μM pre-incubated Aβ_42_ sample on a piece of mica and allowing it air-dried before observation. The incubation conditions for the pre-incubation of 25 μM Aβ_42_ samples with or without inhibitors were the same as in the ThT assays.

### Cell viability assay

The MTT method was employed for cell viability assay^52,53^ by using SH-SY5Y as a human neuronal cell model^54,55^. A total of 5 × 10^3^ of SH-SY5Y cells (80 μL) were cultured in DMEM/F12 medium containing 10% FBS, 1% glutamic acid, 1% penicillin and streptomycin at 37 °C under 5% CO_2_ for 24 h in a 96-well plate. To assess the cytotoxicity induced by Aβ_42_, 25 μM Aβ_42_ solutions (20 μL) pre-incubated with or without an inhibitor for 16 h were added to the wells. The cells were incubated for another 48 h, and then 10 μL of 5.5 mg/mL MTT in the PBS solution was added into each well and incubated for another 4 h. The suspension was centrifuged at 1,500 rpm for 10 min to remove the supernatant. Then, 100 μL DMSO was added to dissolve the formazan, followed by shaking at 150 rpm for 10 min. The cell viability was calculated using the absorbance value at 570 nm measured by a microplate reader (TECAN Infinite, Salzburg, Austria). The absorbance of the sample treated without cells was subtracted and the cell survival treated with the PBS only was set as control to normalize other data for comparison. Six replicates were conducted and the averaged data with standard deviations are reported. Student’s t-test (unpaired parametric test) was calculated for statistical comparisons to analyze the variance and p < 0.05 or less was considered to be statistically significant.

### Data availability

The datasets generated during and/or analyzed during the current study are available from the corresponding author on reasonable request.

## Electronic supplementary material

Supplementary Materials
