# Supplementary material for: Nanogels of dual inhibitor-modified hyaluronic acid function as a potent inhibitor of amyloid β-protein aggregation and cytotoxicity
Source: Sci Rep. 2018 Feb 22;8:3505. doi: 10.1038/s41598-018-21933-6 (PMC5823891; doi:10.1038/s41598-018-21933-6)
Supplement: Supplementary file 1 — Supplementary Materials [file 41598_2018_21933_MOESM1_ESM.docx]

Supplementary Materials

**Nanogels of dual inhibitor-modified hyaluronic acid function as a potent inhibitor of amyloid β-protein aggregation and cytotoxicity**

**Zhiqiang Jiang^1^, Xiaoyan Dong^1,^*, Xin Yan^1^, Yang Liu^2^, Lin Zhang^1^, Yan Sun^1,^***

^1^ Department of Biochemical Engineering and Key Laboratory of Systems Bioengineering of the Ministry of Education, School of Chemical Engineering and Technology, Tianjin University, Tianjin 300072, China

^2^ Department of Biology & Guangdong Provincial Key Laboratory of Marine Biotechnology, College of Science, Shantou University, Shantou, Guangdong 515063, China

∗ Corresponding authors at:

Tel.: +86 22 27404981; Fax: +86 22 27403389. E-mail address: d_xy@tju.edu.cn (Xiaoyan Dong); ysun@tju.edu.cn (Yan Sun).

**Table S1.** Properties of EHA conjugates and assembled hydrogels

| EHA | SD_E_ | Zeta potential  (mV) ^a^ |
| --- | --- | --- |
| EHA1  EHA2  EHA3 | 1.67  4.63  12.7 | -39.9±9.9  -52.8±4.0  -60.2±4.8 |

^a^ Zeta potentials of EHAs in the PBS solution at 37℃.

**Table S2.** CEHA conjugates and the properties of the assembled hydrogels

| CEHA | SD_E_ | SD_C_ | Size  (nm) ^a^ | Zeta potential  (mV) ^b^ |
| --- | --- | --- | --- | --- |
| CEHA1  CEHA2  CEHA3  CEHA4  CEHA5  CEHA6  CEHA7  CEHA8  CEHA9  CEHA10  CEHA11 | 1.67  1.67  1.67  4.63  4.63  4.63  12.7  12.7  12.7  3.32  5.05 | 1.32  2.51  3.54  1.29  2.54  3.47  1.31  2.55  3.49  2.55  3.87 | 207.2 ± 14.8  170.8 ± 19.7  121.4 ± 16.3  175.7 ± 15.6  167.3 ± 14.5  119.6 ± 20.8  155.3 ± 11.0  130.5 ± 10.9  111.2 ± 13.2  168.4 ± 20.0  107.6 ± 13.2 | -27.0±1.9  -26.8±1.8  -24.1±2.4  -17.3±1.7  -17.0±0.9  -16.3±1.1  -15.0 ± 1.3  -15.1 ± 2.0  -14.6 ± 1.4  -19.2 ± 3.2  -16.1 ± 2.5 |

^a^ Average hydrodynamic sizes of CEHAs in the PBS solution at 37℃.

^b^ Zeta potentials of CEHAs in the PBS solution at 37℃.


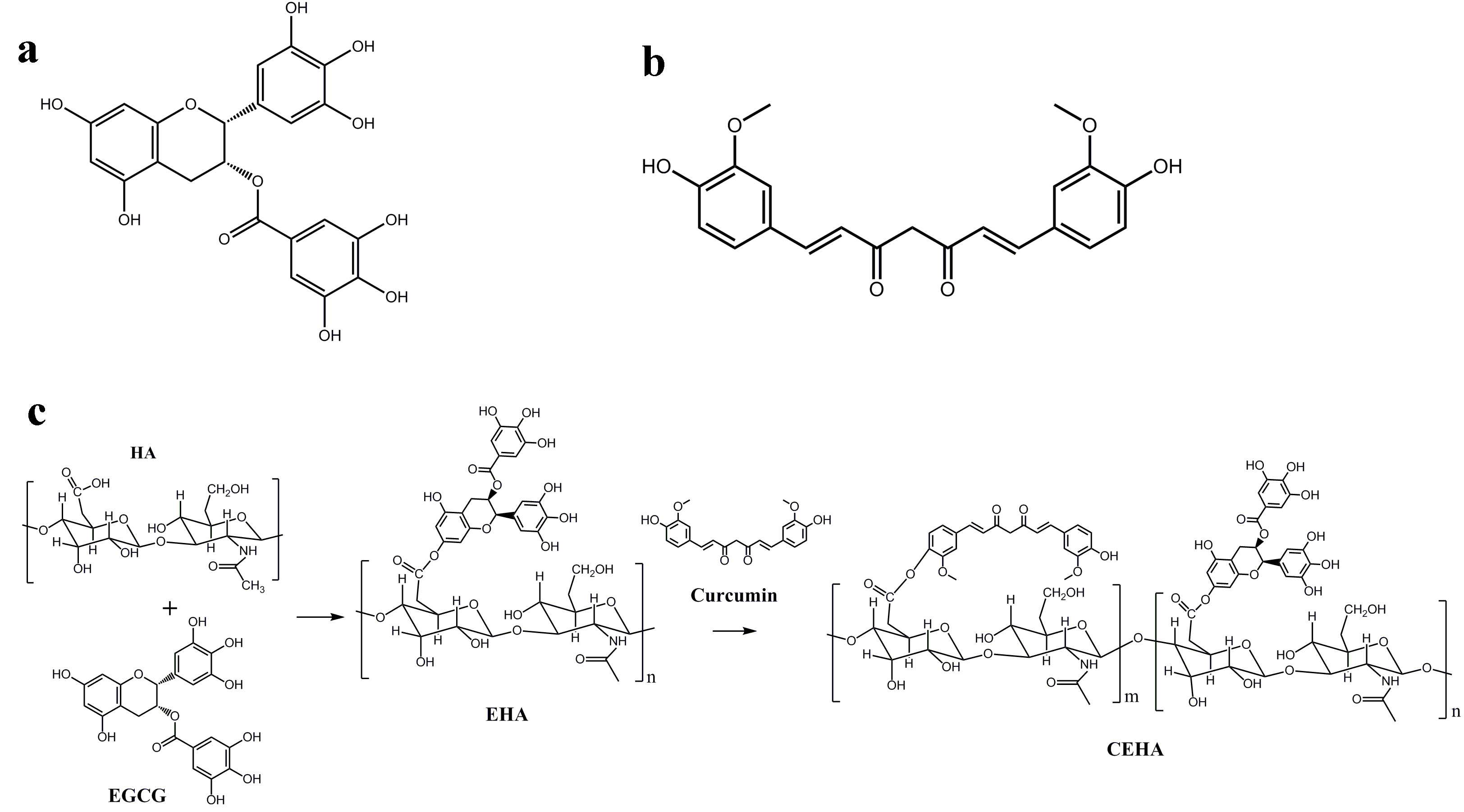


**Figure S1.** Structural formula of (a) EGCG and (b) curcumin, and (c) schematic diagram for the syntheses of EHA and CEHA.


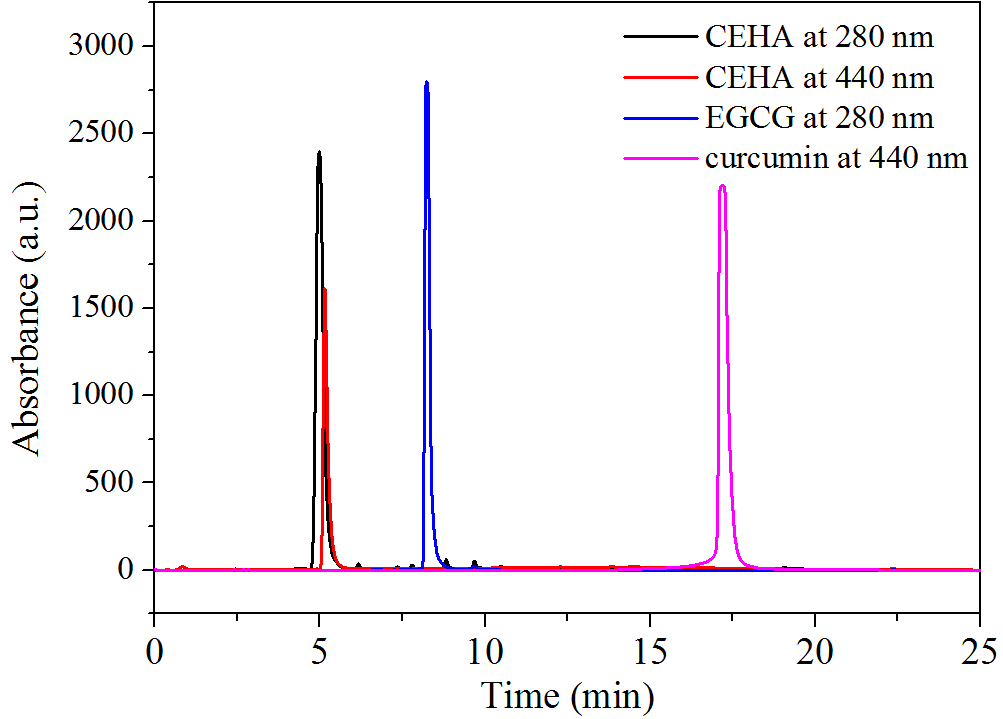


**Figure S2.** RP-HPLC chromatograms of CEHA, EGCG and curcumin.


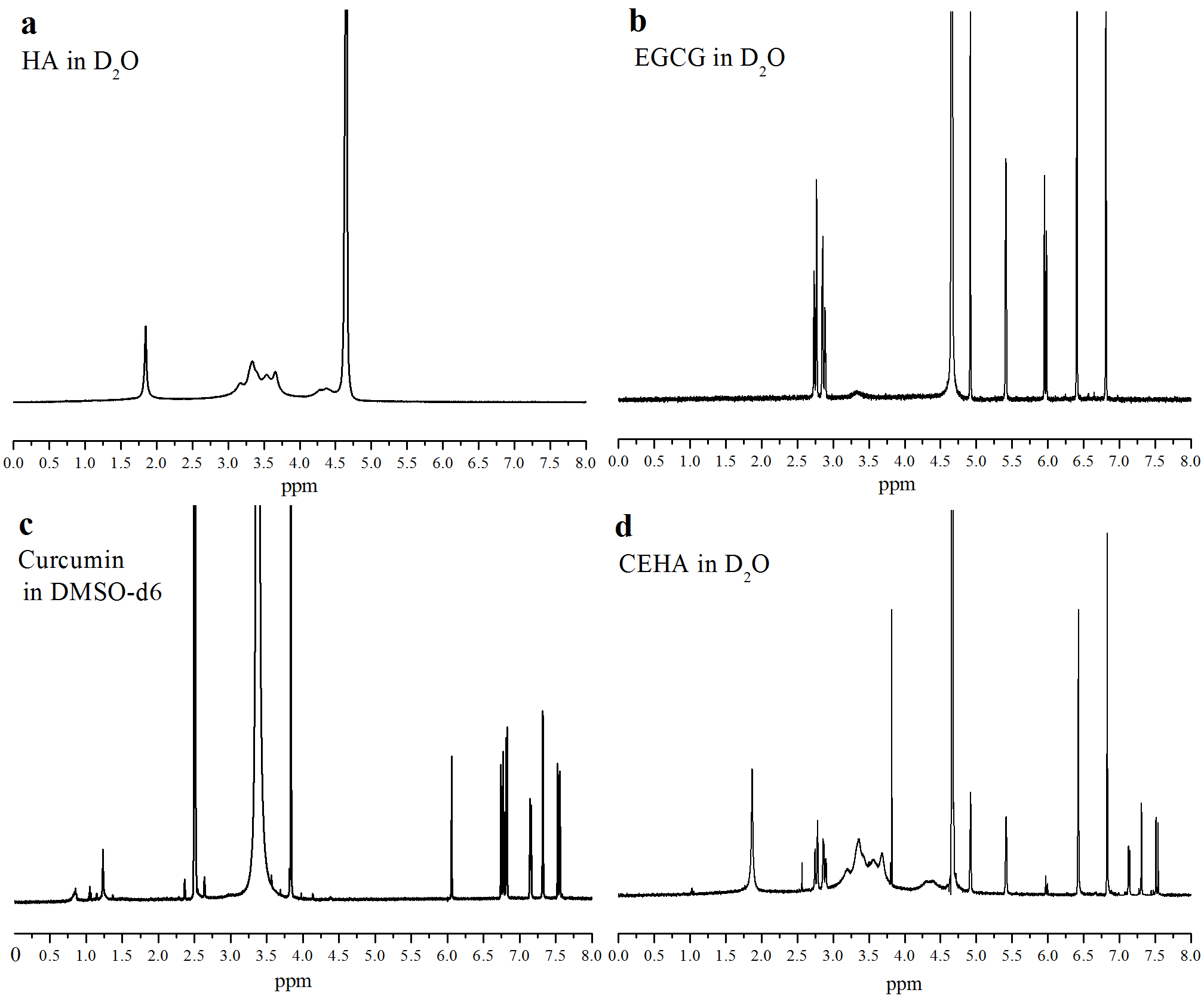


**Figure S3.** NMR spectra of (a) HA, (b) EGCG, (c) curcumin and (d) CEHA in deuterated solvents.


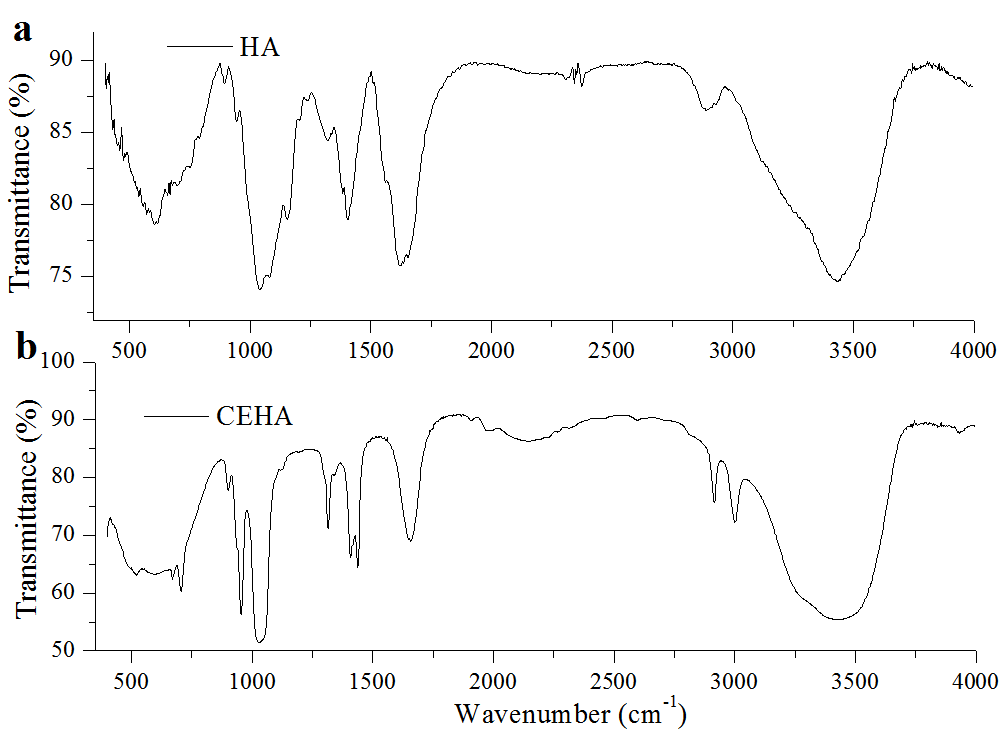


**Figure S4.** FTIR spectra of (a) HA and (b) CEHA.


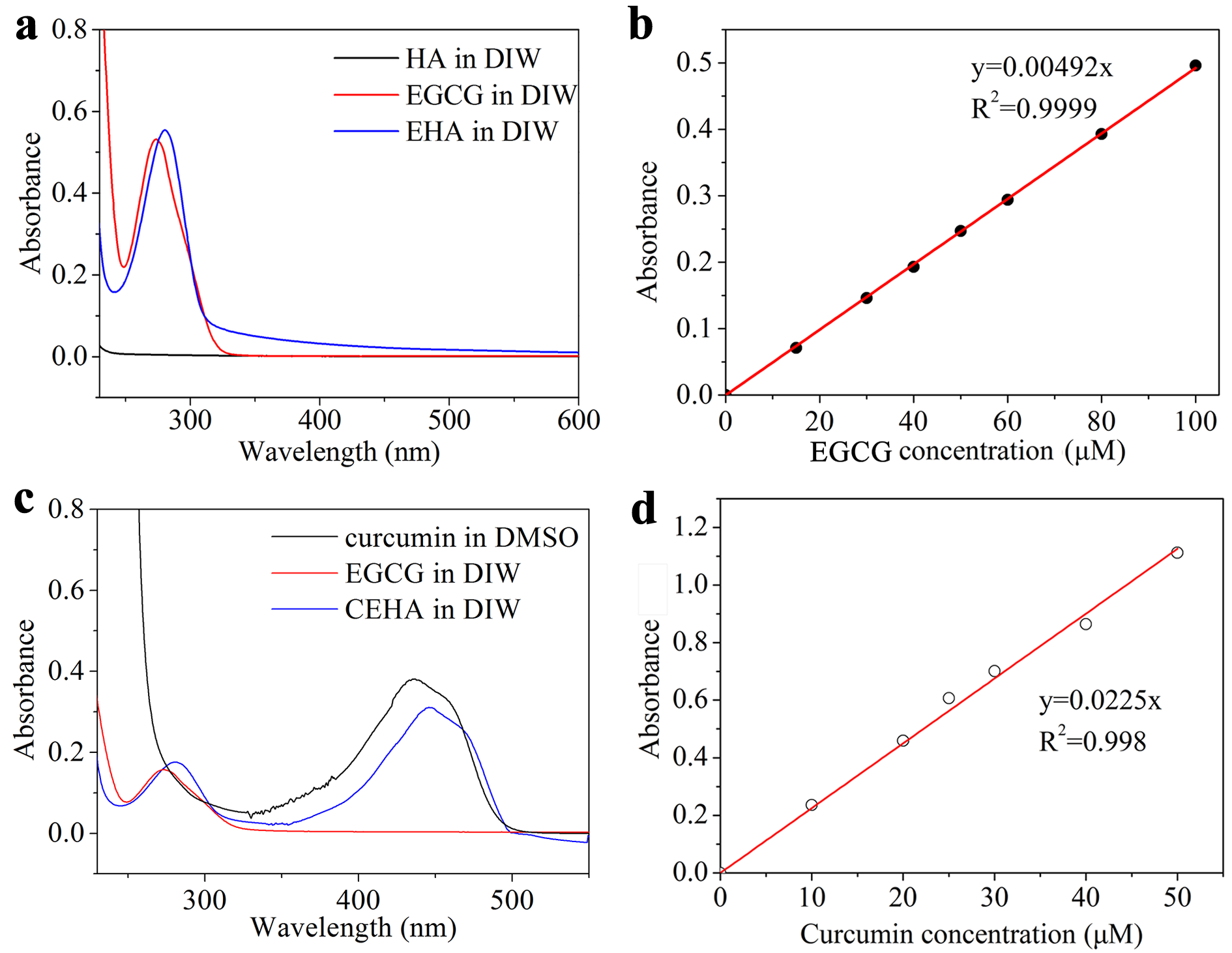


**Figure S5.** (a) UV-visible spectra of HA, EGCG and EHA in deionized water (DIW); (b) Calibration curve of EGCG in DIW determined at 280 nm; (c) UV-visible spectra of CEHA and EGCG in DIW and curcumin in DMSO; (d) Calibration curve of curcumin determined at 440 nm. The curcumin solution was prepared by dissolving it in DMSO, and pure DMSO was used as the background of the visible absorbance measurement.


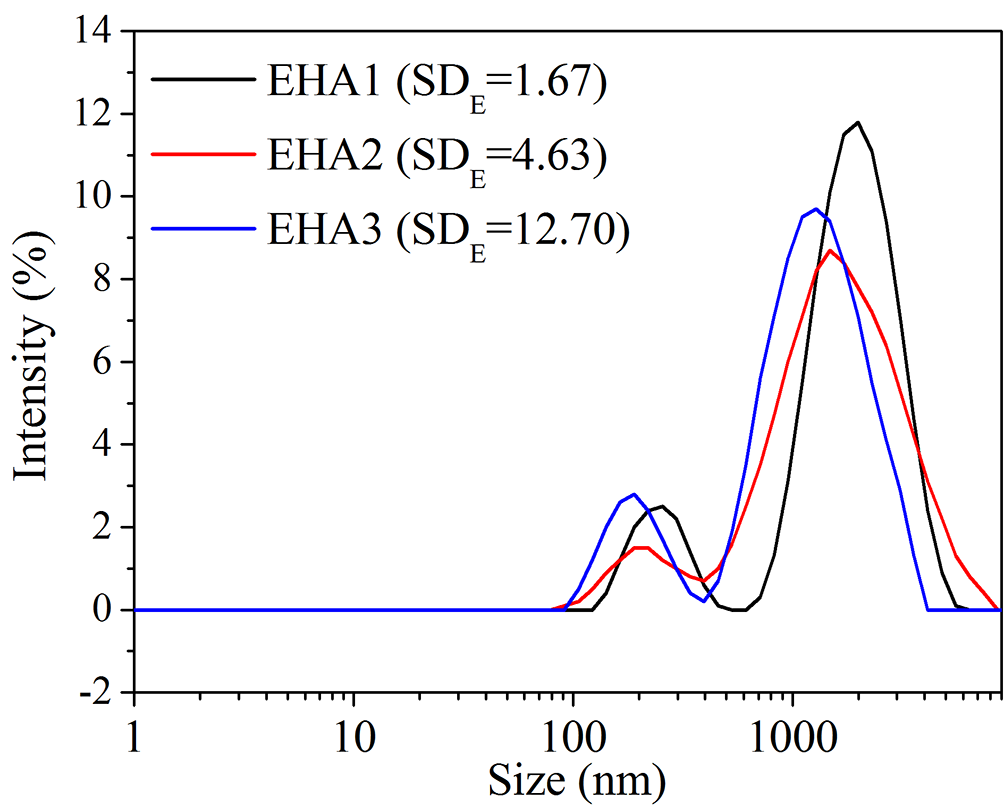


**Figure S6.** Size distributions of the three EHAs determined by DLS in PBS solution at 37℃.


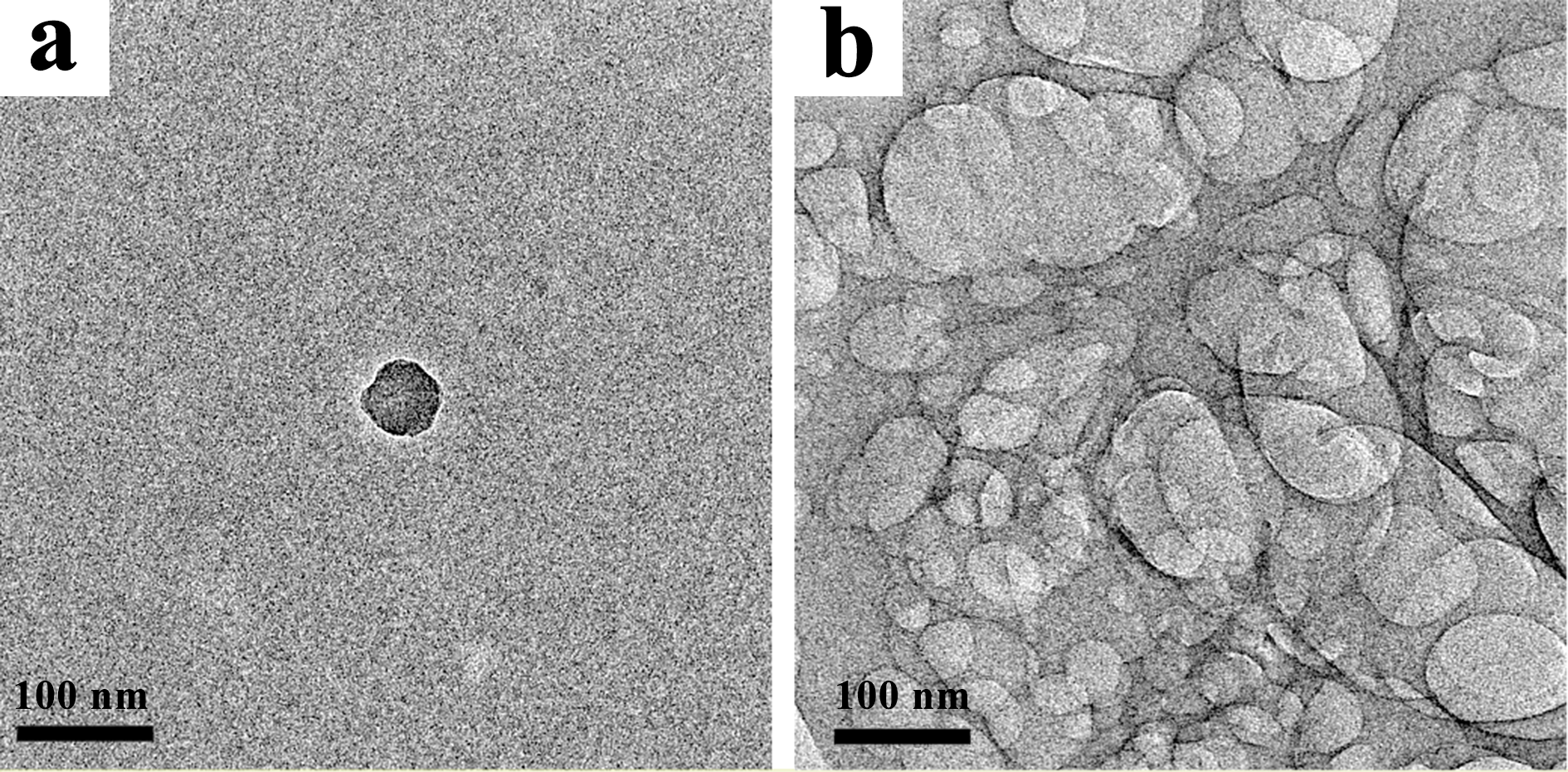


**Figure S7.** TEM images of EHA3. Less single NPs were observed as in (a), but more disperse-structured gels were observed for this EHA with the highest SD_E_ values (b).


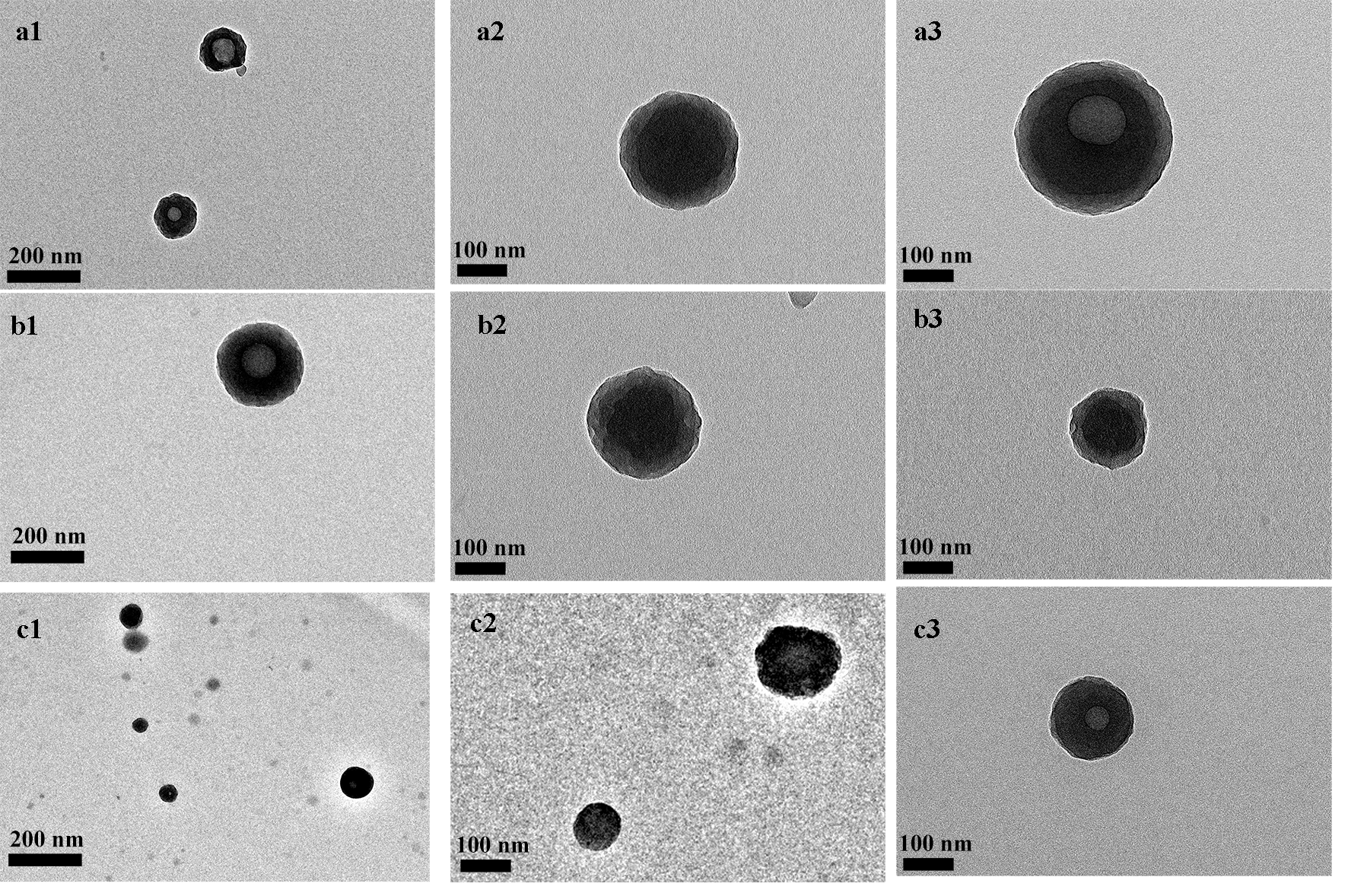


**Figure S8.** TEM images of CEHA4 (a1, a2, a3), CEHA5 (b1, b2, b3) and CEHA6 (c1, c2, c3).

**
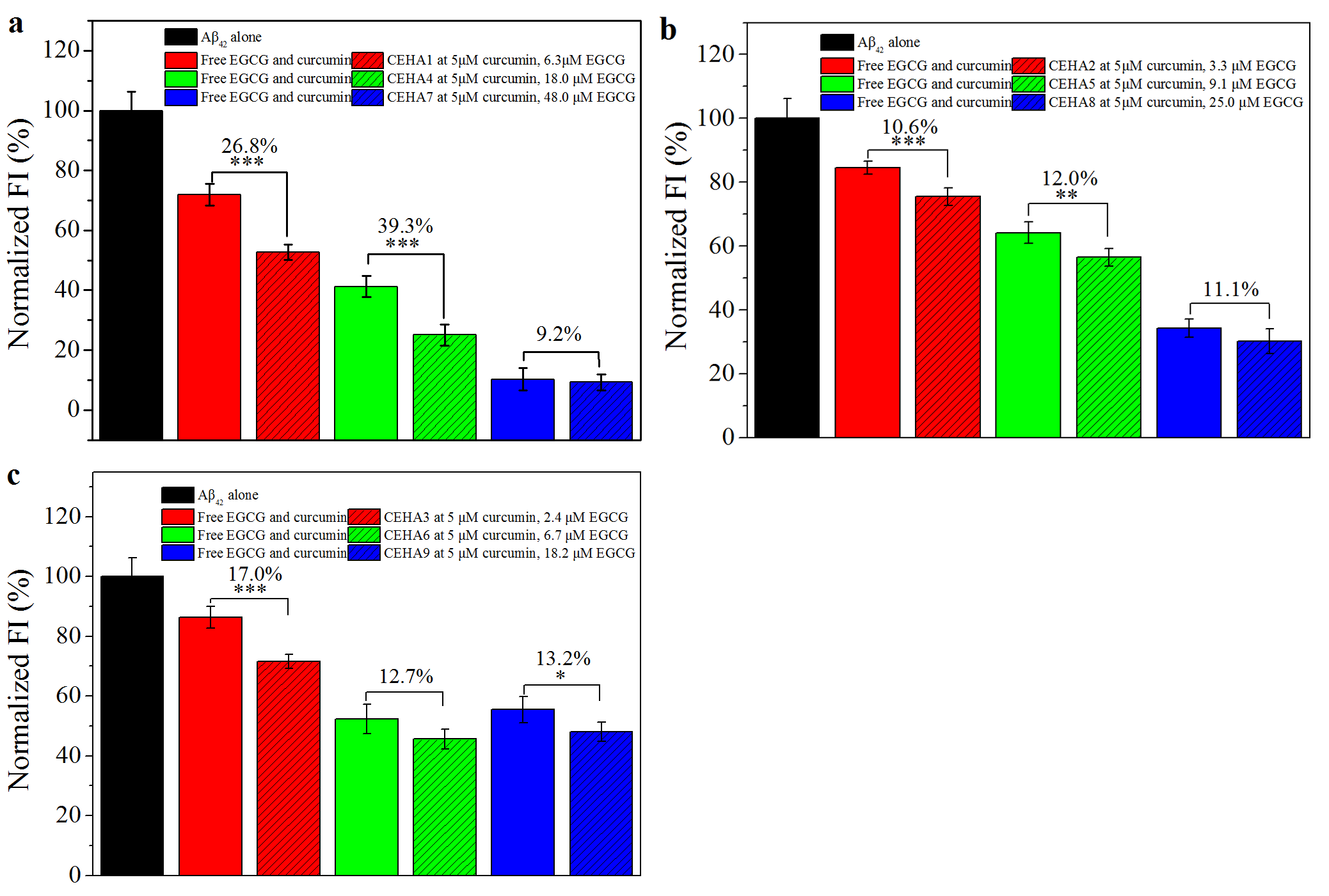
**

**Figure S9.** ThT FI of Aβ_42_ incubated with different CEHAs by comparison with the mixture of free EGCG and curcumin at the same inhibitor concentrations at each group. Curcumin concentration was fixed at 5μM with varying EGCG concentrations. (a) CEHA1, CEHA4, CEHA7 (SD_C_=1.3); (b) CEHA2, CEHA5, CEHA8 (SD_C_=2.5); (c) CEHA3, CEHA6, CEHA9 (SD_C_=3.5). The figures in percentage over the columns stand for the RPs of the ThT FI for the CEHA groups (slash bars) as compared to those for the corresponding free EGCG-curcumin groups (solid bars). ***, p < 0.001, **, p < 0.01 and *, p < 0.05 compared to the free inhibitor groups.


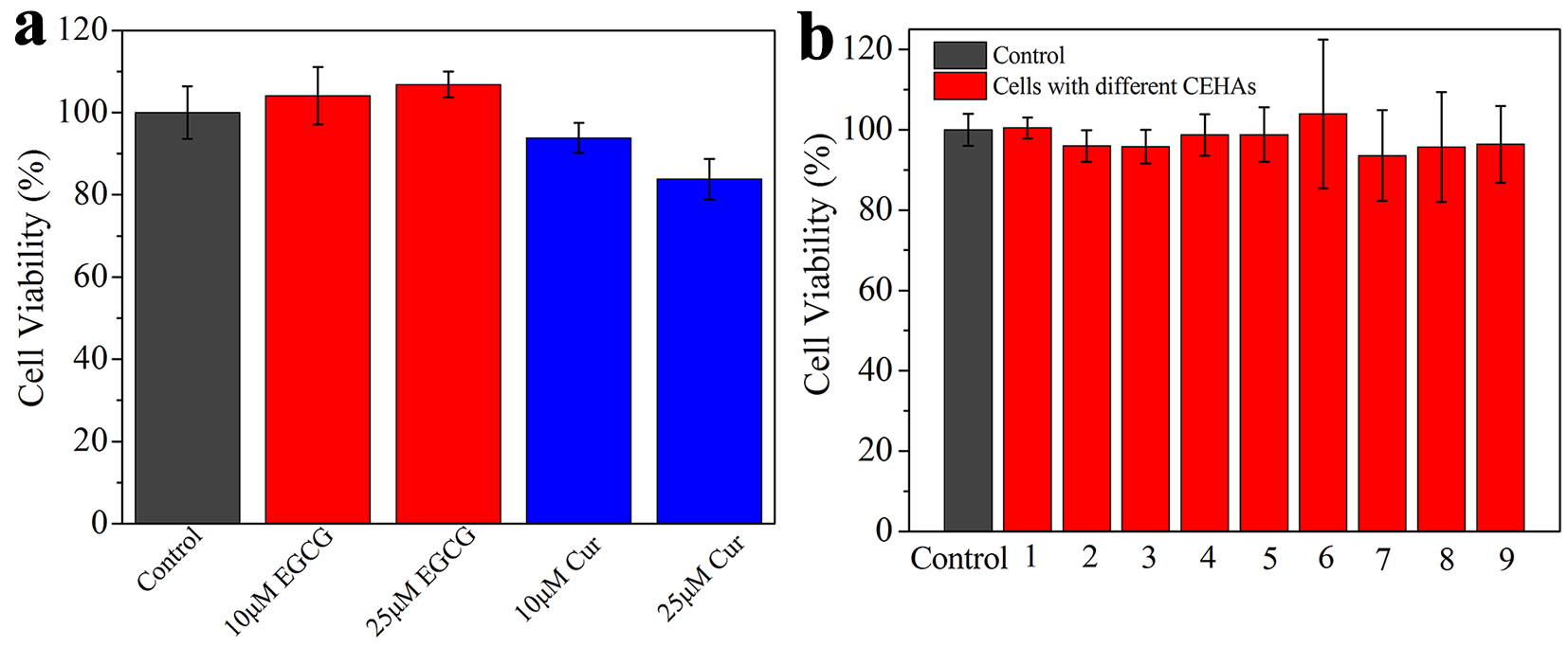


**Figure S10.** The cell viability of SH-SY5Y cells incubated with (a) free EGCG and curcumin of different concentrations, and (b) the nine CEHA conjugates at 25 μM curcumin.


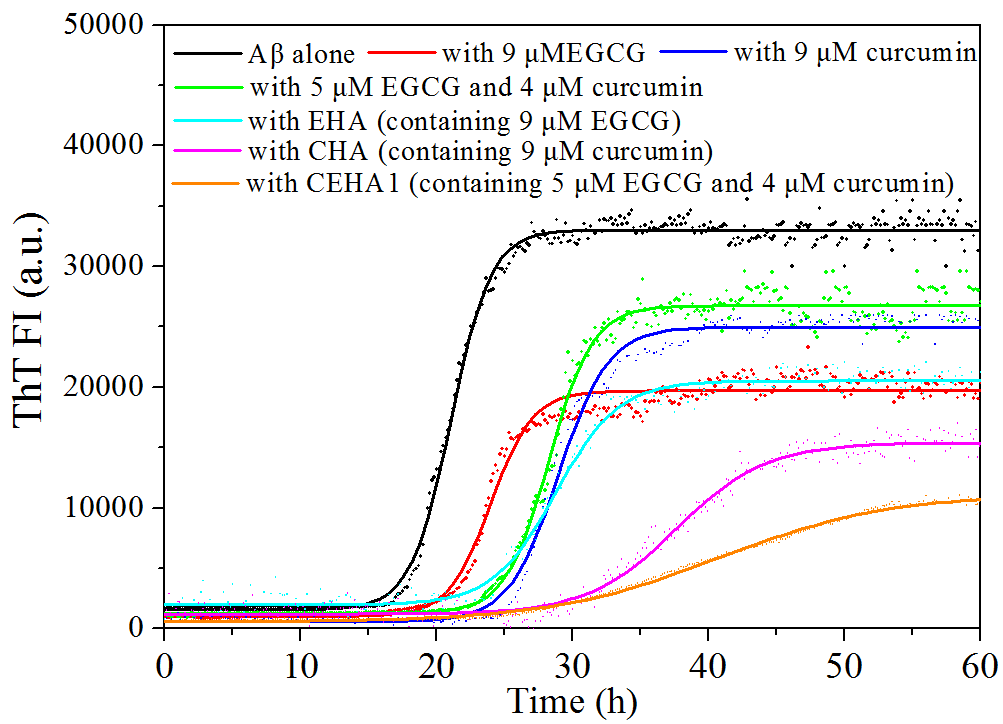


**Figure S11.** Kinetic growth curves of Aβ_40_ incubated with or without different inhibitors measured by microplate reader.
